# Items

**Published:** 1905-04

**Authors:** 


### ITEMS.

The New York Pharmacal Association, Yonkers, has issued recently a panel chart in colors, to illustrate Hæckel's theory of evolution. It will be found of especial value to the student, by means of which he will be enabled to fix in his mind the epochs of the several geological eras, the flora and fanna belonging to each, and the descent of man through each by an ingenious ancestral tree. The chart will be sent to any physician who may not have received it, if a request be made to the New York Pharmacal Association.

The Denver Chemical Company, New York, proprietors of antiphlogistine, has published an artistic booklet dealing with substitutions and the substitutor in an original fashion. The cover page bears a life-like portrait of the original substitutor, clothed in his official robes. Anyone who may not have seen it should apply to the publishers for a copy.

Battle & Company, Saint Louis, have sent out the fifth of their series of twelve illustrations of intestinal parasites. The entire group will be sent free to physicians on application to the publisher.

